# Effect of Breeding Heavier Romney Ewe Lambs at Seven Months of Age on Lamb Production and Efficiency over Their First Three Breeding Seasons

**DOI:** 10.3390/ani11123486

**Published:** 2021-12-07

**Authors:** Emmanuelle Haslin, Rene A. Corner-Thomas, Paul R. Kenyon, Emma J. Pettigrew, Rebecca E. Hickson, Steve T. Morris, Hugh T. Blair

**Affiliations:** School of Agriculture and Environment, Massey University, Palmerston North 4474, New Zealand; r.corner@massey.ac.nz (R.A.C.-T.); p.r.kenyon@massey.ac.nz (P.R.K.); e.pettigrew@massey.ac.nz (E.J.P.); r.hickson@massey.ac.nz (R.E.H.); s.t.morris@massey.ac.nz (S.T.M.); h.blair@massey.ac.nz (H.T.B.)

**Keywords:** energy requirements, feed efficiency, predicted pasture intake, total lamb weaning weight

## Abstract

**Simple Summary:**

This experiment examined the impact of breeding heavier ewe lambs on lamb production and the ability of a ewe to produce a high number and heavier lambs (i.e., efficiency) over the first three breeding seasons. Two groups of ewe lambs were bred at seven months of age at an average live weight of either 47.9 ± 0.36 kg (*n* = 135) or 44.9 ± 0.49 kg (*n* = 135). Ewe live weight, number of lambs born and weaned, and lamb live weights were recorded, and ewe efficiency was calculated for each ewe. The weight and number of lambs at weaning over the three-year period did not differ between treatments, however, when data were pooled, heavier ewe lambs at breeding had a greater number and lamb weaning weight over the three-year period. Breeding heavier ewe lambs had no effect on ewe efficiency. There was a positive relationship between ewe lamb breeding live weight and their mature weight. These results suggest that breeding heavier ewe lambs increased the total lamb production over the three-year period, however, it had no effect on ewe efficiency. Before final recommendations can be made, ewe lifetime performance and longevity of heavier ewe lambs at breeding are required.

**Abstract:**

This experiment examined the effect of breeding heavier ewe lambs on lamb production and their efficiency over their first three breeding seasons. Two groups of ewe lambs were bred at seven months of age at an average pre-breeding live weight of either 47.9 ± 0.36 kg (heavy; *n* = 135) or 44.9 ± 0.49 kg (control; *n* = 135). Ewe live weight, number of lambs born and weaned, and lamb live weight were recorded until 39 months of age, and efficiency was calculated for each ewe. Although the number and lamb weaning weight did not differ between treatments over three years, when data were pooled, heavier ewe lambs at breeding weaned a greater number of lambs over the three-year period. The total lamb weaning weight over the three-year period increased by 2% for each additional kilogram at ewe lamb breeding. Breeding heavier ewe lambs had no effect on efficiency. These results suggest that although breeding heavier ewe lambs had a positive effect on lamb production over the three-year period, it had no effect on efficiency. Before final recommendations can be made, lifetime performance and longevity to five years of age of heavier ewe lambs at breeding are required.

## 1. Introduction

Breeding ewe lambs at seven months of age can increase farm productivity, the number of lambs born per ewe in her lifetime, and enable early selection of replacement ewes [1]. A significant driver of the reproductive performance of ewe lambs is ensuring they are of suitable live weight at breeding, with the positive relationship between live weight at breeding and reproductive performance being well documented [1,2,3,4]. Kenyon et al. [1] recommended that, in New Zealand, a minimum live weight target should be 40 kg at breeding for Romney-type ewe lambs. To date, studies have focused on the effects of breeding ewe lambs on lifetime performance and efficiency compared to those not bred until 17–19 months of age [5,6,7,8,9], rather than the influence of their ewe lamb breeding live weight per se.

Thomas and Berger [10] investigated the impacts of greater prepubertal growth rates of dairy ewe lambs on their subsequent reproductive performance. They reported no effect of greater growth rates (245 g/d vs. 327 g/d) on fertility or the number of lambs born to four years of age [10]. In Haslin et al. [11], the heavy and control Romney ewe lambs showed a three kg difference in live weight at breeding (47.9 ± 0.36 vs. 44.9 ± 0.49 kg, respectively). It was observed that the heavy group had greater reproductive performance than the control group at first breeding [11], however, during their second and third breeding seasons, reproductive performance did not differ [12]. The long-term consequences of breeding Romney ewe lambs at a heavier live weight on reproductive efficiency over multiple breeding seasons is unknown.

The efficiency of a ewe is related to her ability to wean a large number of heavy lambs relative to her live weight [13,14]. Sheep efficiency can be defined as the ratio of the total weaning weight of her litters and her live weight at breeding. While feed efficiency can be defined as the ability of a ewe to convert feed into the live weight of lambs weaned [15], it can be estimated by dividing the total kg of lambs weaned by the estimated ewe feed intake over a period of time. A major component of efficiency, therefore, is the ewe mature live weight, as a heavier ewe will have greater maintenance requirements compared to a lighter ewe [15,16]. Based on similar lamb production, a lighter ewe would, therefore, be more efficient than a heavier ewe [14,17]. Thus, it is of interest to determine if ewe lamb breeding live weight and later live weight affect lifetime efficiency.

The current experiment had three objectives. Firstly, to examine the effect of a heavier live weight at the first breeding at seven months of age, on productive performance and the efficiency of ewes over their first three breeding seasons. Secondly, to determine the impact of successfully weaning a lamb as a ewe lamb on ewe performance and efficiency over their first three breeding seasons. Thirdly, to examine the impact of breeding heavier ewe lambs on feed requirements and mature live weight at 39 months of age.

## 2. Materials and Methods

The experiment was conducted at Massey University‘s Riverside farm (latitude: 40°50′35 S, longitude: 175°37′55 E), 10 km north of Masterton, and at Keeble farm (latitude: 41°24′03 S, longitude: 175°35′51 E), 5 km south of Palmerston North, New Zealand. The experiment and all animal handling procedures were approved by the Massey University Animal Ethics Committee (MUAEC17/16).

### 2.1. Experimental Design

The experimental design has been previously described in detail by Haslin et al. [11] and [12]. Briefly, at weaning (3 January 2018; approximately 86 days of age; d86), 270 twin-born Romney ewe lambs were allocated to one of two treatments (heavy and control) using a stratified random sampling procedure so that weaning live weight did not differ between treatments (28.6 kg ± 0.16). Post-weaning, the heavy group (*n* = 135) was preferentially fed with a greater amount of concentrate than the control group until breeding at seven months of age (10 May 2018; d213) achieving an average live weight of 47.9 ± 0.36 kg, and the control group (*n* = 135) weighed an average of 44.9 ± 0.49 kg at d213. From d213 until the weaning of their third set of lambs (d1159), ewes from the heavy and the control groups were managed as one cohort. All ewes were bred at an average of 7 (d213), 19 (d567; 29 April 2019), and 30 months of age (d913; 9 April 2020) for 34 days. Each year, their pregnancy status and the number of fetuses were determined by trans-abdominal ultrasound at pregnancy diagnosis (d301, d660, and d995). All ewes were shorn by commercial operators at d337, d638, d810, and d962. Pre-lambing (d351, d701, and d1050), pregnant ewes were allocated to lambing paddocks based on the number of fetuses (1, 2, or 3) and the period of conception (first or second 17-day period of the breeding period). Ewes from the heavy and control groups were present in all lambing paddocks. Each year, non-pregnant ewes were grazed separately from pre-lambing (d351, d701, and d1050) to weaning of the progeny (d465, d800, and d1159) and re-joined the flock after each weaning. During the lambing periods, ewes were checked twice daily to identify newborn lambs. Lambs were ear marked and their tails were removed in early lactation, at approximately 25 days of age (d394, d746, d1095) and were weaned at approximately 100 (d465), 78 (d800), and 91 days of age (d1159). Ewes that failed to conceive in a given year, aborted, or whose entire litter died were not removed from the experiment to allow for determination of long-term performance.

### 2.2. Feeding Management

The difference in live weight at breeding at seven months of age (d213) between the control and the heavy groups was achieved by offering differing herbage allowances prior to breeding on either lucerne (*Medicago sativa* L.) or a ryegrass (*Lolium perenne* L.) and white clover-based (*Trifolium repens* L.) sward and cereal-based concentrate feed allowances [11]. Prior to the first breeding (d213), both treatments grazed lucerne (heavy from d86 to d191 & control from d86 to d186) and then ryegrass/white clover-based sward (heavy from d191 to d213 & control from d186 to d213), and were both offered a cereal-based concentrate feed (CP 10.5%, NDF 17.6%, ADF 7.1%, ME 12.8 MJ/kg DM). The cereal-based concentrate feeds were provided due to severe drought conditions experienced during this period at Riverside farm. The control group was offered 200 g/ewe lamb/day of cereal-based concentrate for 43 days (d119 to d162) and the heavy group was offered 200 g/ewe lamb/day for 68 days (d94 to d162) and 300 g/ewe lamb/day for 51 days (d162 to d213). Individual ewe intakes were not measured. 

From d213 onwards, ewes were grazed on ryegrass/white clover pasture under commercial New Zealand grazing conditions. In 2018, ewe lambs were offered pre-grazing pasture mass of an average of 920 ± 44 kg DM/ha and 1265 ± 58 kg DM/ha in pregnancy (d213 to d351) and lactation (d351 to d465), respectively [11]. In 2019, due to dry summer conditions post-weaning, ewes were offered 0.9 kg/ewe/day of grass bailage from d504 to d524 reduced to 0.5 kg/ewe/day from d524 to d592 in addition to ryegrass/white clover pasture [12]. Ewes were also offered approximately 200 g/ewe/day of a cereal-based supplement from d537 to d611 [12]. The pre-grazing pasture mass in 2019 and 2020 was on average 2209 ± 167 kg DM/ha and 1814 ± 144 kg DM/ha in pregnancy (d567 to d701 and d913 to d1050), and 1592 ± 63 kg DM/ha and 1433 ± 586 kg DM/ha in lactation (d701 to d800 and d1050 to d1159), respectively [12].

### 2.3. Animal Measurements

**Figure 1:**
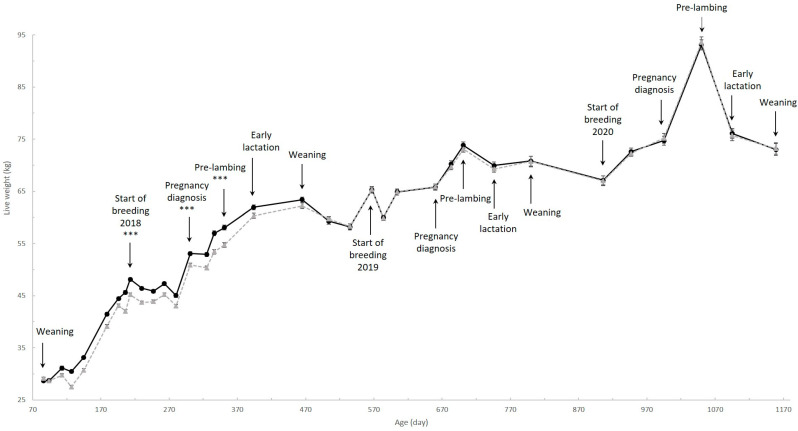
Live weight (± SEM) of all ewes in the heavy (black solid line) and the control (grey dotted line) groups from their weaning (d86) to the weaning of their third set of lambs at three years of age (d1159). *** indicates treatment differences at *p* < 0.001.

Unfasted live weights of ewes were recorded at d86 (weaning), d94, d113, d127, d145, d179, d196, d206, d213 (ewe lamb breeding in 2018), d230, d247, d263, d280, d301 (pregnancy diagnosis 2018), d325, d336, d351 (pre-lambing 2018), d394, d465 (weaning 2018), d504, d535, d567 (two-year-old ewe breeding in 2019), d584, d604, d660 (pregnancy diagnosis 2019), d683, d701 (pre-lambing 2019), d746, d800 (weaning 2019), d906 (breeding of three-year-old ewes in 2020), d947, d995 (pregnancy diagnosis 2020), d1050 (pre-lambing 2020), d1095 and d1159 (weaning 2020) (Figure 1). The body condition score of ewes (BCS; [18,19]) was recorded at d213, d247, d351, d394, d465, d567, d601, d660, d746, d800, d995, d1050, d1095, and d1159 [11,12].

At pregnancy diagnosis (d301, d660, and d995), ewes were identified as pregnant or not pregnant and the number of fetuses was recorded. Each year, the number of lambs born, and the number of lambs weaned for each ewe were recorded. Ewe deaths were recorded throughout the three years. During the current experiment, ewes were only removed from the experiment for welfare reasons.

During lambing periods, within 18 h of birth, lambs were identified to their mother, and their date of birth, sex, and birth rank were recorded. Live weights of lambs born in each year were recorded within 18h of birth, at d394, d746, and d1095, and at weaning (d465, d800, and d1159).

### 2.4. Data Management

#### 2.4.1. Ewe Lamb Status

For data analysis, at weaning in 2018 (d465), ewes were retrospectively identified to one of three ewe lamb status categories: ‘weaned a lamb’ when ewes weaned at least one lamb as a ewe lamb, ‘failed to wean a lamb’ when ewe lambs were identified as pregnant and aborted, or whose entire litter died before d465 or ‘not pregnant’ [11].

#### 2.4.2. Number and Live Weight of Lambs

The total number of lambs born, and the total number of lambs weaned over three years (2018, 2019, and 2020) was calculated for each individual ewe. The total birthweight of lambs over the three years was calculated by summing the birthweights of lambs born to each individual ewe from each year. Similarly, the total progeny live weight in early lactation (approximately 25 days of age) and at weaning over the three years was calculated for each ewe by summing the yearly live weights of lambs. Ewes that gave birth to multiple lambs of which one or more lambs died, had the total lamb live weight calculated based on the live weights of the surviving lambs. Ewes that did not conceive in a given year were given a zero for the number of lambs born, weaned, and lamb birth and weaning weights for that year. Ewes that had no surviving lambs in a given year were given a zero for the lamb weaning weight and number of lambs weaned for that year. Ewes that died were removed from the data set from that point onwards.

To determine the effect of ewe lamb status on reproductive performance of ewes, litter weaning weight and the number of lambs weaned in only the second and third year of production were calculated for each individual ewe.

Due to an absence of significant (*p* > 0.10) differences between treatments, live weight at ewe lamb breeding (d213) and their total progeny weaning weight and the total number of lambs weaned were pooled together as one population.

#### 2.4.3. Efficiency

Production efficiency was calculated for each year over the three-year period using the yearly litter weaning weight divided by ewe live weight at the breeding of each year (2018, 2019, and 2020) as per Pettigrew et al. [20].

Ewe live weights were adjusted during pregnancy using the Gompertz equation [21] to calculate conceptus-free live weight based on lambing dates and litter birthweights. A transformational regression was used to fit a polynomial spline curve to the adjusted live weights of each ewe as undertaken by Pettigrew et al. [20]. Spline knots were placed at birth, weaning of the ewe (d86) and thereafter every breeding (d213, d567, and d906), pre-lambing (d351, d701, and d1050), and weaning (d465, d800, and d1159). Second, third, and fourth-order polynomial splines were fitted to ewe adjusted live weights. The best polynomial spline model was selected using the Akaike information criterion (AIC), coefficient of determination (r^2^), coefficient of correlation (r), mean square prediction error (MSPE), root of the mean square prediction error (RMSPE), and relative prediction error (RPE) [22].

The MSPE is an indicator for the error of predicted values relative to the observed values [23], is expressed in kg^2^, and was calculated as follows:

$$\mathrm{MSPE}=\frac{1}{n}\;\sum^{\;}{\left({\mathrm{Actual}}_{i}-{\mathrm{Predicted}}_{i}\right)}^{2}$$

where n is the number of experimental observations; Actual and Predicted are the actual and predicted live weights; i = 1, 2, …, n [23]. 

The RMSPE is the accuracy of prediction, is expressed in kg, and was calculated as follows [22]:

$$\mathrm{RMSPE}=\sqrt{\mathrm{MSPE}}$$

The RPE was calculated as follows [22]:

$$\mathrm{RPE}=\frac{\mathrm{RMSPE}}{{\mathrm{Actual}}_{m}}\times 100$$

where Actual_m_ is the mean actual live weight. The lower the MSPE, RMSPE, and RPE, the more accurate the predictions in the model.

Based on the above criteria, the third-order polynomial spline was selected as the most appropriate model to predict ewe adjusted live weights, as the criteria of third and fourth-order polynomials were very similar, with the fourth order adding little benefit (Table 1). A daily predicted live weight was generated for each individual ewe from their weaning as a lamb (d86), until their death or the end of the experiment (d1159), as undertaken by Pettigrew et al. [20].

As per the study of Pettigrew et al. [20], the daily predicted live weights from the spline models between the start of the treatments (d86) to the weaning of their third litter (d1159) were used to determine the daily energy requirements for each ewe for their maintenance and liveweight change [16]. Litter birth dates and weights were used to determine daily energy requirements for pregnancy [16,21]. Ewes that died between breeding and pregnancy diagnosis were considered as not pregnant and therefore no pregnancy requirement was calculated until death. Ewes who were identified as pregnant with one or more fetuses and died between pregnancy diagnosis and lambing were given an average date of birth and lamb birthweight for their treatment for that year. Ewes that were identified as pregnant at pregnancy diagnosis, but did not lamb, were assumed pregnant and to have aborted just before the expected lambing date and were given an average lambing date and lamb birthweight for their treatment for that year. Ewe lactation energy requirements were calculated with the energy required to produce milk, which was based on ewe milk yield and days of lactation [21] and the proportion of their litter’s energy requirement provided by pasture, which depends on lamb energy requirement for maintenance and growth to weaning [16,21]. Ewe milk yield was modelled from Peart et al. [24] based on the number of lambs reared and week of lactation, as undertaken by Pettigrew et al. [20]. Lamb energy requirements met by milk or pasture were calculated based on birth weight and date, average daily gain between birth and weaning, and weaning weight and date [16,21]. Lamb growth was assumed to be linear between birth and weaning [20]. Lambs that died before weaning (d465, d800, and d1159) or that did not have weights at weaning were assumed to have died at birth, therefore, no lamb or ewe lactation energy requirements were calculated for these lambs. Equations for the estimation of ewe daily energy requirements are presented in Appendix A.

The estimated total energy requirement between d86 and d1159 was calculated for each ewe, and based on this, a total predicted pasture intake (kg DM) was also estimated. The total predicted pasture intake was based on the total estimated energy requirement for each ewe and the average energy content of white clover/ryegrass pastures over a year in the Central North Island of New Zealand (10.3 ± 0.51 MJME/kg DM; [25]). A feed efficiency value for each individual ewe between d86 and d1159 (total progeny weaning weight/total predicted pasture intake) was calculated based on the total progeny weaning weight and the total predicted pasture intake over the three breeding seasons.

#### 2.4.4. Survival

All ewes alive at the end of the experiment (d1159) were censored at that date for the experiment survival analysis. In this experiment, ewes were only culled for welfare reasons, therefore a hypothetical culling policy was retrospectively imposed on ewes to simulate ewe retention in the flock with commercial farming conditions, as undertaken by Pettigrew et al. [20]. In the hypothetical culling policy, in 2018, ewes identified as non-pregnant at d301 were culled, as suggested by Kenyon [26] as a means of increasing flock productivity. Ewe lambs whose entire litter died before weaning (d465) were not culled. In the hypothetical culling policy, at two and three years of age, ewes were culled if they did not conceive at pregnancy diagnosis (d660 and d995) or if all their lambs died by day 25 of lactation (d746 and d1095) or by weaning (d800 and d1159), which is a common farm practice in New Zealand [27].

### 2.5. Statistical Analyses

All statistical analyses were conducted using SAS v9.4 (SAS Institute Inc., Cary, NC, USA). Ewe live weights have been reported in Haslin et al. [11] for live weight in 2018 as ewe lambs and in Haslin et al. [12] for live weight in 2019 and 2020 as two- and three-year-old ewes.

The yearly number of lambs born, and the number of lambs weaned per ewe presented for breeding were analysed with generalized linear models assuming Poisson distributions and using log transformations. The models included treatment (control vs. heavy), ewe lamb status (weaned a lamb vs. failed to wean a lamb vs. not pregnant) and year as fixed effects, and ewe as a random effect.

The yearly litter birth weight, litter weight in early lactation (approximately 25 days of age), litter weaning weight per ewe bred, and yearly production efficiency were analysed with linear mixed models. Treatment, ewe lamb status, and year were included as fixed effects, and ewe was included as a random effect.

The total number of parities, lambs born, and lambs weaned over the three years were analysed using generalized linear models with Poisson distributions and using log transformations. Treatment and ewe lamb status were included as fixed effects. The total progeny weaning weight over three breeding seasons, and the estimated feed efficiency between d86 and d1159 were analysed with linear models including treatment and ewe lamb status as fixed effects.

The combined number of lambs weaned in the second and third years only was analysed with a generalized linear model with a Poisson distribution and a log transformation. Ewe lamb status was included as a fixed effect in the model. The combined litter weaning weight of lambs in the second and third year of production was analysed with a linear model including ewe lamb status as a fixed effect.

Survival analysis of ewes was carried out using the age at which ewes were removed prior to d1159 or died. In addition, a hypothetical culling policy was applied to ewes, retrospectively to determine a predicted ewe retention rate. Survival analyses of the trial ewes and the hypothetical culling policy were compared between treatments and ewe lamb statuses.

Due to an absence of significant (*p* > 0.10) differences between treatments, live weight at ewe lamb breeding (d213) and their three-year-old live weight at weaning (d1159) were pooled. A linear regression model was constructed to examine the relationship between the live weight of ewe lambs at d213 and their mature live weight at d1159, irrespective of treatment. Ewe lamb status and pregnancy rank (single- or twin-bearing) as a ewe lamb at d301 were also included in the regression model.

A linear regression model was constructed to examine the relationship between ewe live weight at d213 and the total progeny weaning weight over their first three years of production, irrespective of treatment.

## 3. Results

### 3.1. Ewe Live Weight and BCS

Differences in live weight and BCS of ewe lambs between the heavy and the control group were reported in detail in Haslin et al. [11] and the BCS and live weight differences of two- and three-year-old ewes in Haslin et al. [12] (Figure 1). Briefly, ewes in the heavy group were heavier (*p* < 0.001) than those in the control group from d213 to d351 after which time they did not differ (*p* > 0.10; [11,12]) (Figure 1). Ewes in the heavy group had a greater BCS (*p* < 0.01) than those in the control group at d213 and d301 after which time they did not differ (*p* > 0.10; [11,12]).

Liveweight differences between ewe lamb status (i.e., weaned a lamb vs. conceived but failed to wean a lamb vs. not pregnant) at two and three years of age were reported in detail in Haslin et al. [12]. Briefly, ewes that weaned a lamb as a ewe lamb were lighter (*p* < 0.05) than ewes that conceived but failed to wean a lamb or were not pregnant from d567 to d701, but did not differ (*p* > 0.10) after that [12]. The live weight of ewes that failed to wean a lamb and ewes that were not pregnant as a ewe lamb did not differ (*p* > 0.10) at any time point [12]. Ewes that weaned a lamb as a ewe lamb had a lower BCS (*p* < 0.05) than ewes that were not pregnant as a ewe lamb at d567 and d660 [12]. Ewe lamb status had no effect on BCS between d746 and d1159 [12].

### 3.2. Lamb Production

#### 3.2.1. Treatment Effect

The yearly number of lambs born and weaned per ewe across the three years did not differ (*p* > 0.10) between the heavy and control groups (Table 2). Based on ewe lamb status, ewes that weaned a lamb as a ewe lamb gave birth to a greater (*p* < 0.001) number of lambs per year across the three years than those not pregnant as a ewe lamb. The number of lambs born per year to ewes that failed to wean a lamb at d465 was intermediate and did not differ (*p* > 0.10) from either ewes that weaned a lamb or that were not pregnant as a ewe lamb (Table 2). Ewes that weaned a lamb as a ewe lamb weaned a greater (*p* < 0.001) number of lambs per year than ewes that were not pregnant or failed to wean a lamb as a ewe lamb, which did not differ (*p* > 0.10; Table 2).

The yearly litter weight at birth, early lactation, and weaning per ewe presented for breeding over the three-year period did not differ (*p* > 0.10) between the heavy and control group (Table 3). Ewes that weaned a lamb as a ewe lamb had greater (*p* < 0.001) yearly litter birth weights across the three years than ewes that failed to wean a lamb, which had a greater (*p* < 0.001) litter birth weight than ewes that were not pregnant (Table 3). Ewes that weaned a lamb as a ewe lamb had greater (*p* < 0.01) yearly litter weights in early lactation and at weaning over the three-year period than ewes that failed to wean a lamb and were not pregnant as a ewe lamb, which did not differ (*p* > 0.10; Table 3).

The total number of parities, lambs born, lambs weaned, and total progeny weaning weight over the three-year period did not differ between treatments (*p* > 0.10; Table 4). Ewes that were not pregnant as a ewe lamb had a lower (*p* < 0.01) total number of parities and number of lambs born over three years than both ewes that weaned a lamb or that failed to wean a lamb as a ewe lamb, which did not differ (*p* > 0.10). Ewes that weaned a lamb as a ewe lamb weaned 40% and 84% more lambs (*p* < 0.05) over three years than ewes that were not pregnant and failed to wean a lamb as a ewe lamb respectively, which in turn did not differ (*p* > 0.10). The total progeny weaning weight over three years was greater (*p* < 0.05) for ewes that weaned a lamb as a ewe lamb than ewes that were not pregnant, which was greater (*p* < 0.05) than ewes that failed to wean a lamb. The combined number of lambs weaned in the second and third years of production did not differ (*p* > 0.10) between ewe lamb status. The combined number of lambs weaned in the second and third year by ewes that as a ewe lamb either weaned a lamb was 2.60 (2.34–2.88), did not become pregnant was 2.61 (2.32–2.94) or that failed to wean a lamb was 2.04 (1.55–2.68). Ewes that failed to wean a lamb as a ewe lamb had a lower (*p* < 0.05) combined litter weaning weight in the second and third year of production than ewes that weaned a lamb or were not pregnant as a ewe lamb, which did not differ (*p* > 0.10; 53.6 ± 6.01 vs. 68.2 ± 2.55 and 71.3 ± 2.96, respectively).

#### 3.2.2. Effect of Live Weight at Ewe Lamb Breeding Irrespective of Treatment

**Figure 2:**
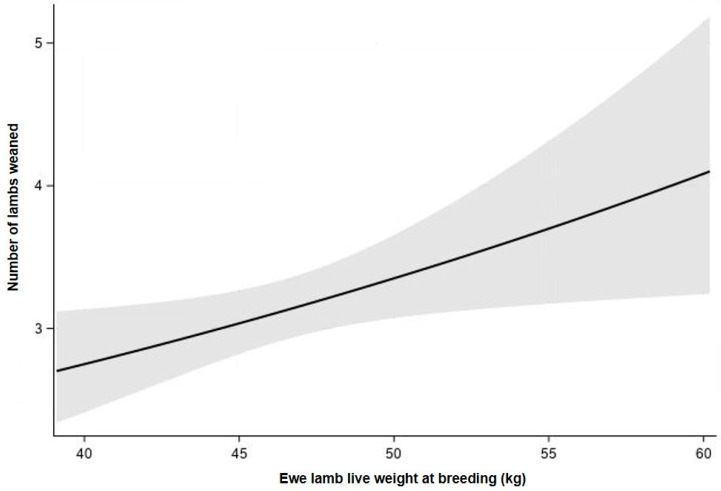
Number of lambs weaned over the three-year period in relation to ewe lamb live weight at breeding (d213) (log predictions (solid line) and 95% confidence intervals shown (grey area)).

**Figure 3:**
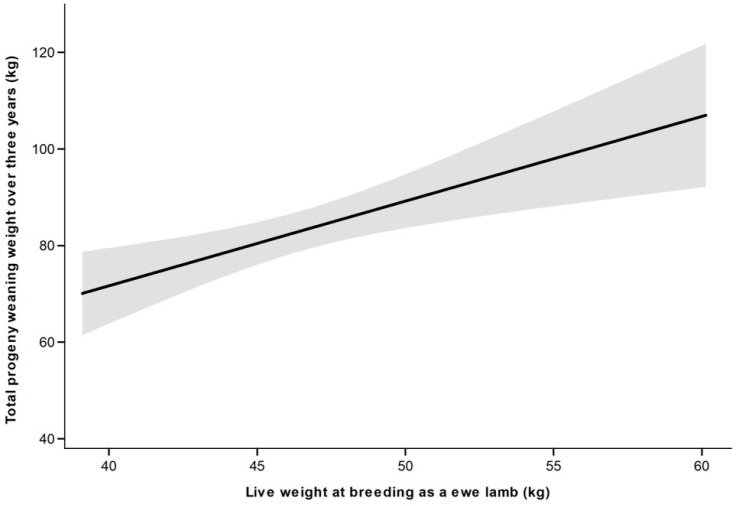
Total progeny weaning weight (kg) over the first three years of production in relation to ewe lamb live weight at breeding (d213). Predictions and 95% confidence intervals shown (r^2^ = 0.044).

As there was no difference (*p* > 0.10) in the total number of lambs weaned over the three-year period between treatments, data from the two treatments were pooled. Ewe lamb live weight at d213 was positively associated (*p* < 0.05) with the total number of lambs weaned and the total progeny weaning weight over the three-year period (Figure 2 and Figure 3). The total progeny weaning weight over the first three breeding seasons increased by 2% for every additional kilogram at ewe lamb breeding (Figure 3).

### 3.3. Efficiency

The yearly production efficiency (litter weaning weight/ewe breeding live weight per year) did not differ (*p* > 0.10) between treatments (Table 3). Ewes that weaned a lamb as a ewe lamb had a greater (*p* < 0.01) yearly production efficiency than ewes that were not pregnant, which was greater (*p* < 0.01) than ewes that failed to wean a lamb as a ewe lamb (Table 3).

The total predicted pasture intake and estimated feed efficiency over the first three years did not differ between treatments (*p* > 0.10; Table 4). Ewes that weaned a lamb as a ewe lamb had a greater (*p* < 0.01) total predicted pasture intake over the first three years than both ewes that were not pregnant and those that conceived but failed to wean a lamb as a ewe lamb (Table 4). Ewes that weaned a lamb as a ewe lamb showed 29% greater feed efficiency (*p* < 0.001) between d86 and d1159 than ewes that were not pregnant and 79% greater feed efficiency (*p* < 0.001) than ewes that failed to wean a lamb as a ewe lamb (Table 4). 

### 3.4. Ewe Survival to Three Years of Age (d1159)

**Figure 4:**
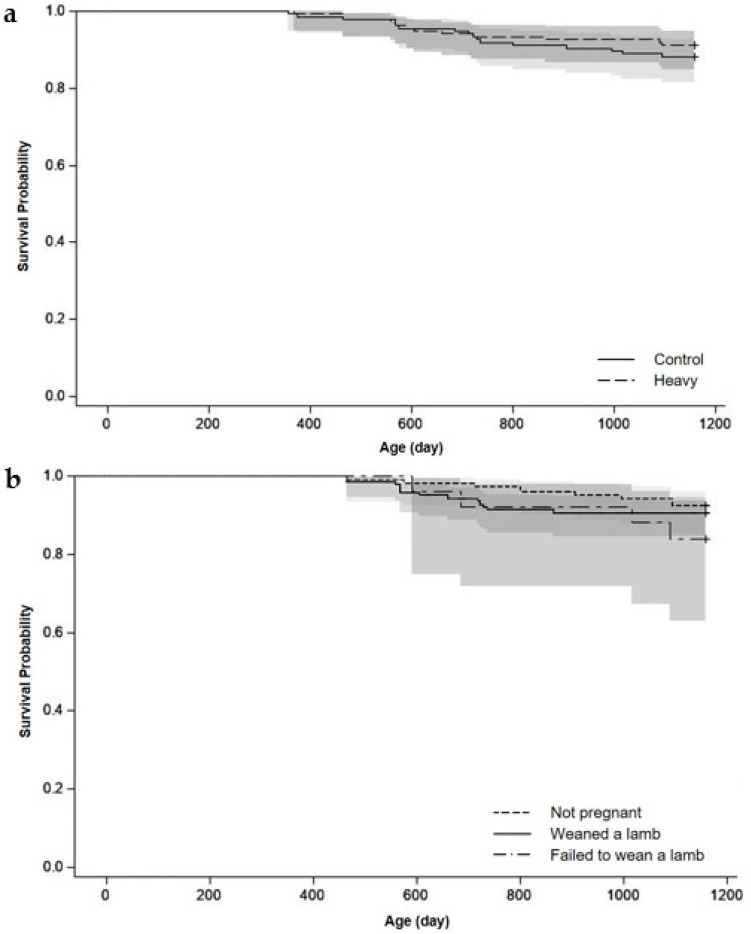
Actual survival curves and 95% confidence interval (grey area) of the ewes based on (**a**) treatments (control or heavy) and (**b**) ewe lamb status (not pregnant or weaned a lamb or failed to wean a lamb) for the three years of the experiment until 1159 days of age. Ewes were only culled for welfare reasons.

**Figure 5:**
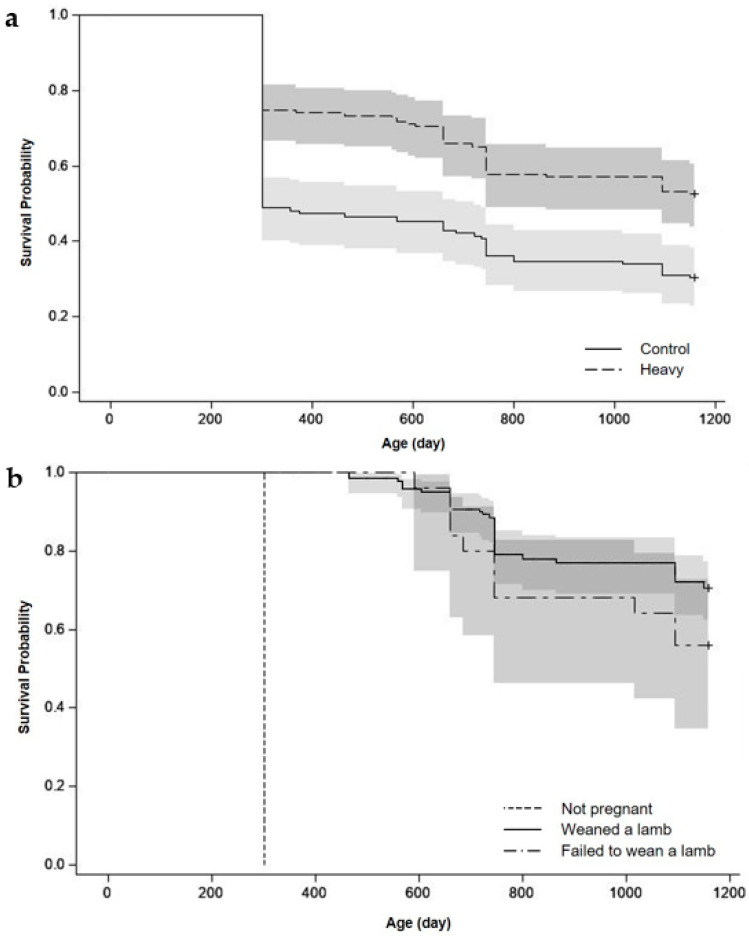
Predicted retention curves and 95% confidence interval (grey area) of ewes in the flock based on (**a**) treatments (control or heavy) and (**b**) ewe lamb status (not pregnant or weaned a lamb or failed to wean a lamb) for the three-year period of the experiment at 1159 days of age with the hypothetical culling policy: ewe lambs that were not pregnant were culled at pregnancy diagnosis (d301) and ewe lambs whose lambs died were not culled, and at two and three years of age, non-pregnant ewes at pregnancy diagnosis (d660 and d995) and ewes whose lambs died before weaning (d800 and d1159) were culled.

The actual survival of ewes to d1159 did not differ (*p* > 0.10) between ewe lamb treatments and ewe lamb status (Figure 4a,b). The predicted retention of ewes in the flock from the heavy group, based on a hypothetical culling policy, was greater than the predicted retention of ewes from the control group (*p* < 0.001; Figure 5a). The predicted retention to d1159 of ewes in the flock that weaned a lamb as a ewe lamb was greater (*p* < 0.001) than the predicted retention of ewes that failed to wean a lamb as a ewe lamb (Figure 5b).

### 3.5. Relationship between Ewe Lamb Breeding Live Weight and Ewe Mature Weight

**Figure 6:**
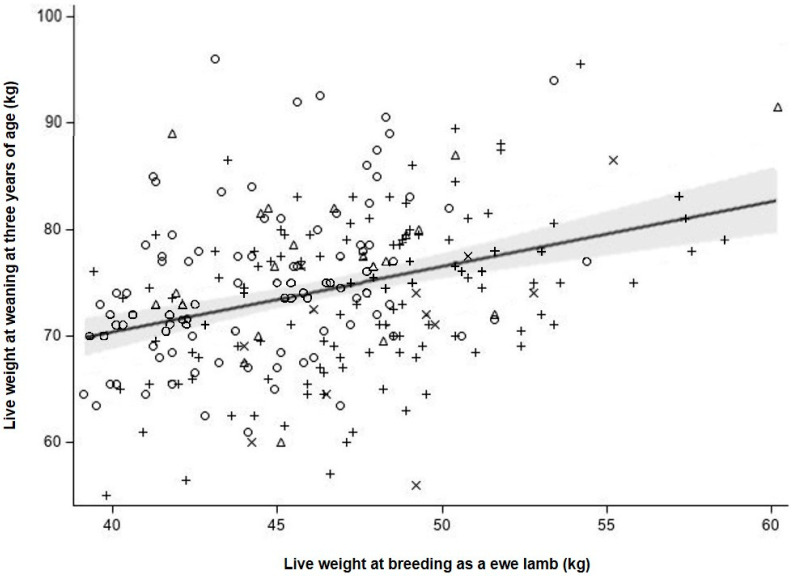
Ewe live weight at weaning at 39 months of age (d1159) in relation to their live weight at their first breeding at seven months of age (d213) displaying their status (failed to wean a lamb (triangles) or not pregnant (circles)) and pregnancy rank (single-bearing (plus) or twin-bearing (crosses)) as a ewe lamb. Predictions and 95% confidence intervals are shown.

As there was no difference (*p* > 0.10) in live weight at d1159 between treatments, data of the two treatments were pooled. Ewe live weight at weaning at three years of age (d1159) was positively associated (*p* < 0.01) with ewe live weight at their first breeding (d213) (r^2^ = 0.113; ewe live weight at d1159 = 45.5 (±5.23) + 0.62 (±0.11) ewe live weight at d213; Figure 6). For each 1 kg increase in breeding live weight of ewe lambs (d213), mature live weight at 39 months of age (d1159) increased by 0.62 ± 0.112 kg (Figure 6).

## 4. Discussion

### 4.1. Effect of Heavier Ewe Lamb Live Weight on Lamb Production and Efficiency

The first objective of the current experiment was to examine the effects of a heavier breeding live weight, as a ewe lamb, on ewe performance and efficiency over their first three breeding seasons. Treatment had no effect on lamb production over the three years, however, when data were pooled, a heavier live weight at breeding was positively associated with the total lambs produced. The addition of ewe lamb status in the statistical models created a confounding effect with treatment and obscured any effect of the treatment. In Haslin et al. [11], the heavy group had greater fertility than the control group, therefore, there was a greater proportion of ewes from the heavy group that ‘weaned a lamb as a ewe lamb’ and a greater proportion of ewes from the control group that were ‘not pregnant’. The large range of ewe lamb breeding live weight within each treatment (heavy 41.0 to 60.2 kg and control 39.1 to 57.6 kg) may have limited the identification of an effect of a heavier live weight and explained the differences in results between treatment and pooled data. These results suggest that there is a positive effect of heavier live weight at ewe lamb breeding on the total number of lambs produced over the first three breeding seasons, which was due to improved ewe lamb reproductive performance.

Yearly production efficiency (litter weaning weight/ewe breeding live weight) and feed efficiency (total progeny weaning weight/estimated pasture intake) did not differ between treatments over the three-year period. The control group was lighter at ewe lamb breeding and weaned fewer lambs than the heavy group [11]. The three-kilogram difference in ewe lamb breeding live weight between treatments, combined with the greater lamb production of ewe lambs in the heavy group, resulted in production efficiency being similar between treatments. At two and three years of age, lamb production and ewe breeding live weight did not differ between treatments [12]. Over the first three breeding seasons, the total progeny weaning weight and estimated pasture intake did not differ between treatments, therefore, there was no difference in the feed efficiency.

Yearly production efficiency in the present experiment was similar to that reported by Pettigrew et al. [28] of ewes born as either a single or twin to a ewe lamb during their first two productive years (0.39 kg lamb weaned/kg at ewe breeding per year). Yearly values, however, were lower than those reported by Pettigrew et al. [20] for ewes born as twins to mature ewes over eight years of production (0.70 kg lamb weaned/kg at ewe breeding). The differences between these studies might be explained by the eight-year period considered by Pettigrew et al. [20], the age at first breeding (seven or 18 months of age), and dam age (ewe lambs or mature ewes) which can affect body size.

The actual survival rate of the ewes did not differ between treatments over the three-year period. When a hypothetical culling policy was applied, however, the heavy group showed greater predicted ewe retention to 39 months of age in the flock than the control group. The hypothetical culling policy consisted of the culling of non-pregnant ewes at pregnancy diagnosis and two- and three-year-old ewes whose lambs died before weaning, which is a policy commonly employed on commercial New Zealand farms. The lower fertility of the control group compared to the heavy group as ewe lambs, which was mainly driven by live weight at ewe lamb breeding [11], explained this difference in ewe predicted retention in the flock.

Heavier live weight at ewe lamb breeding had a positive effect on total lamb production, which was driven by the greater reproductive performance of heavier ewe lambs during their first breeding season [11]. There was, however, no effect of ewe live weight on production efficiency and feed efficiency over their first three breeding seasons. Predicted ewe retention within the flock to 39 months of age was improved for ewe lambs bred at a heavier live weight. Breeding heavier ewe lambs may also impact ewe lifetime production and efficiency and, therefore, needs to be further examined before final recommendations can be made to farmers. Ewe performance should be followed to at least five or six years of age when culling for age traditionally occurs in New Zealand.

### 4.2. Impact of Successfully Weaning a Lamb as a Ewe Lamb on Subsequent Production and Efficiency

Ewes that weaned a lamb as a ewe lamb had greater total lamb and yearly production and feed efficiency over their first three breeding seasons than the two other groups (i.e., failed to wean a lamb and not pregnant). This is consistent with Baker et al. [6] who reported that ewes that lambed as a ewe lamb had a greater weaning rate and lamb weaning weight than those that did not, up to four years of age. Kenyon et al. [7], however, reported that lambing as a ewe lamb had no effect on lamb weaning weight at three years of age. In the current experiment, ewes that weaned a lamb as a ewe lamb were lighter at two-year-old breeding than the two other groups [12], however, they produced heavier litters than the other two groups, which explained the greater production efficiency of ewes that had weaned a lamb. Ewes that weaned a lamb as a ewe lamb had the greatest total progeny weaning weight and had similar pasture intake to ewes that failed to wean a lamb, which explained their greater feed efficiency compared to the two other groups. When comparing the production in their second and third year, ewes that weaned a lamb as a ewe lamb did not differ from ewes that were not pregnant as a ewe lamb. This finding indicates that ewes that weaned a lamb as a ewe lamb did not perform any better than ewes that failed to become pregnant and the difference in production between these two ewe lamb statuses was due to the greater reproductive performance in the first year of production. Ewe survival did not differ between ewe lamb status, however, if a hypothetical culling policy had been applied, the retention in the flock of ewes that weaned a lamb was greater than the predicted retention of ewes that failed to wean a lamb to 39 months of age.

Ewes that failed to wean a lamb as a ewe lamb had the lowest production and feed efficiency over the three-year period. In addition, when considering only the second and third year of production, ewes that failed to wean a lamb had lower lamb weaning weight than those that weaned a lamb or were not pregnant as a ewe lamb, indicating that ewes that failed to wean a lamb had lower performance than ewes that weaned a lamb or were not pregnant as a ewe lamb in the subsequent years of production. These results are consistent with Amer et al. [29] who reported that ewes whose entire litter died prior to weaning had lower lamb and litter weaning weight during the following lambing compared to ewes that successfully weaned a lamb. Griffiths et al. [30] also reported that the risk of losing the entire litter at two years of age was greater for ewes whose entire litter had died during their first lactation compared to ewe lambs that successfully weaned a lamb. Fogarty et al. [31], however, reported that lamb production of ewes that did not lamb as a ewe lamb, and ewes that lambed and lost their lambs, did not differ over the first three breeding periods. In that study, ewes that were identified as pregnant, but did not lamb were not included in the analyses. In the current experiment, ewes that aborted were included in the analysis and likely explained the difference in production. While not statistically different, ewes that failed to wean a lamb had lower yearly litter weaning weight than ewes that had not been pregnant as a ewe lamb, and were three-kilograms heavier than ewes that were not pregnant [12]. Collectively, these differences explain why yearly production efficiency was lower in ewes that failed to wean a lamb than the two other ewe groups. Similarly, ewes that failed to wean a lamb had the lowest feed efficiency and total progeny weaning weight but had similar total pasture intake compared to the two other ewe groups. In addition, ewes that failed to wean a lamb were less likely to survive to 39 months of age than ewes that weaned a lamb as a ewe lamb.

In summary, ewes that weaned a lamb as a ewe lamb were more efficient on a per kg of lamb weaned per kg of dry matter eaten basis than ewes that were not pregnant which in turn were more efficient than ewes that failed to wean a lamb. The results suggest that farmers should cull ewe lambs whose entire litter dies prior to weaning in order to improve flock performance and efficiency. A limitation of this experiment, however, was the low number of ewe lambs that failed to wean a lamb (<30), therefore, these results should be interpreted with caution. Further investigations are needed using a larger number of ewes to determine the consequences of failing to wean a lamb as a ewe lamb.

### 4.3. Relationship between Ewe Lamb Breeding Live Weight and Ewe Live Weight at 39 Months of Age

There was a positive relationship between ewe lamb live weight at breeding and their live weight at 39 months of age, indicating that there was a carry-over effect of greater breeding live weights. Ewe mature live weight is one of the primary components of ewe efficiency [15,17,32]. A heavier mature ewe would, therefore, result in greater maintenance energy requirements and overall feed demand [16,33]. To be as efficient, a heavier mature ewe would need to produce either more or heavier lambs to compensate for the additional feed needed to meet her energy requirements [17,33,34]. Heavier live weight at breeding is known to increase ewe reproductive performance [33,35], therefore, there is value in following the ewes in this experiment until they are culled for age at five years of age to examine whether the efficiency of heavier ewes is affected.

## 5. Conclusions

Breeding ewe lambs at heavier weights had a positive effect on the total lamb production to weaning at 39 months of age, however, there was no effect on production and feed efficiency over the first three breeding seasons. Ewes that successfully weaned a lamb as a ewe lamb had the greatest performance and efficiency, whereas ewes that failed to wean a lamb had the lowest performance and efficiency. There was a positive relationship between ewe lamb breeding live weight and their live weight at 39 months of age. These results suggest that farmers should aim to breed their ewe lambs at heavier live weights to improve their overall performance. Before final recommendations can be made, the performance of this ewe group to five years of age is required.

## Figures and Tables

**Table 1:** Prediction accuracy of second, third, and fourth-order polynomial spline models for the prediction of ewe adjusted live weight over three years.

Model	n	AIC ^a^	r^2^	r	MSPE ^a^ (kg^2^)	RMSPE ^a^ (kg)	RPE ^a^ (%)
Order 2	9118	17,502	0.980	0.990	6.81	2.61	5.06
Order 3	9118	15,925	0.983	0.991	5.73	2.39	4.64
Order 4	9118	15,763	0.983	0.992	5.63	2.37	4.59

n: number of experimental observations; AIC: Akaike information criterion; r^2^: coefficient of determination; r: coefficient of correlation; MSPE: mean square prediction error; RMSPE: root of the mean square prediction error, and RPE: relative prediction error; ^a^ The lower the MSPE, RMSPE, and RPE are, the more accurate the predictions are.

**Table 2:** The effect of treatment (control vs. heavy) and ewe lamb status at d465 ^1^ (weaned a lamb, failed to wean a lamb ^2^, non-pregnant) on least-square means (95% confidence intervals) of the yearly number of lambs born (NLB) and the yearly number of lambs weaned (NLW) per ewe presented for breeding over the first three years of production.

	*n*	NLB/Ewe	NLW/Ewe
Treatments			
Control	135	1.17 (1.04–1.30)	0.84 (0.74–0.96)
Heavy	135	1.25 (1.13–1.39)	0.90 (0.79–1.02)
*p*-value		0.265	0.386
Ewe lamb status at d465 ^1^			
Weaned a lamb	139	1.44 (1.32–1.56) ^b^	1.21 (1.10–1.33) ^b^
Failed to wean a lamb ^2^	25	1.26 (1.03–1.55) ^ab^	0.65 (0.49–0.86) ^a^
Non-pregnant	103	0.98 (0.87–1.10) ^a^	0.84 (0.74–0.95) ^a^
*p*-value		<0.001	<0.001

^a,b^ Means between rows with differing superscripts are different (*p* < 0.05); ^1^ Ewes were retrospectively allocated to the categories at the weaning of their first lambs (d465); ^2^ Was identified as pregnant at d301 but did not wean a lamb at d465.

**Table 3:** The effect of treatment (control vs. heavy) and ewe lamb status at d465 ^1^ (weaned a lamb, failed to wean a lamb, non-pregnant) on least-square means (±SEM) of the yearly litter weight (kg) at birth, early lactation, and weaning per ewe over the three-year period, and the yearly production efficiency (yearly litter weaning weight/ewe live weight at breeding) across their first three years of production.

	*n*	Litter Weight at Birth	Litter Weight in Early Lactation	Litter Weight at Weaning	Yearly Production Efficiency
Treatments					
Control	135	7.97 ± 0.19	13.4 ± 0.50	26.5 ± 0.99	0.420 ± 0.017
Heavy	135	8.11 ± 0.18	13.9 ± 0.47	26.9 ± 0.92	0.422 ± 0.015
*p*-value		0.539	0.334	0.718	0.897
Ewe lamb status at d465 ^1^					
Weaned a lamb	139	9.28 ± 0.16 ^c^	17.9 ± 0.41 ^b^	35.3 ± 0.80 ^b^	0.609 ± 0.014 ^c^
Failed to wean a lamb ^2^	25	8.08 ± 0.37 ^b^	10.6 ± 0.96 ^a^	20.0 ± 1.91 ^a^	0.278 ± 0.032 ^a^
Non-pregnant	103	6.76 ± 0.18 ^a^	12.4 ± 0.47 ^a^	24.7 ± 0.93 ^a^	0.376 ± 0.016 ^b^
*p*-value		<0.001	<0.001	<0.001	<0.001

^a,b,c^ Means between rows with differing superscripts are different (*p* < 0.05); ^1^ Ewes were retrospectively allocated to the categories at the weaning of their first lambs (d465); ^2^ Was identified as pregnant at d301 but did not wean a lamb at d465.

**Table 4:** The effect of treatment (control vs. heavy) and ewe lamb status at d465 ^1^ (weaned a lamb, failed to wean a lamb ^2^, non-pregnant) on least-square means (95% confidence intervals) of the total number of parities, lambs born and weaned over the first three years of production, and least-square means (± SEM) of total progeny weaning weight (kg) over the three-year period, total predicted pasture intake (kg DM) between weaning of the ewe (d86) and the weaning of the third litter (d1159), and estimated feed efficiency (total progeny weaning weight/total predicted pasture intake; kg/kg DM) over the first three years of production.

	*n*	Total Number of Parities	Total Number of Lambs Born	Total Number of Lambs Weaned	Total Progeny Weaning Weight	Total Predicted Pasture Intake	Feed Efficiency
Treatments							
Control	135	2.40 (2.11–2.74)	3.54 (3.18–3.94)	2.60 (2.29–2.97)	72.3 ± 3.19	1484.9 ± 35.5	4.72 ± 0.17
Heavy	135	2.40 (2.12–2.71)	3.85 (3.48–4.24)	2.79 (2.47–3.15)	76.4 ± 3.02	1501.7 ± 33.5	4.85 ± 0.16
*p*-value		0.973	0.202	0.336	0.302	0.698	0.517
Ewe lamb status at d465 ^1^							
Weaned a lamb	139	2.81 (2.55–3.11) ^b^	4.36 (4.03–4.73) ^b^	3.69 (3.38–4.02) ^b^	98.2 ± 2.61 ^c^	1590 ± 29.0 ^b^	6.15 ± 0.14 ^c^
Failed to wean a lamb ^2^	25	2.68 (2.11–3.41) ^b^	3.82 (3.13–4.67) ^b^	2.01 (1.53–2.65) ^a^	52.9 ± 6.13 ^a^	1437 ± 68.1 ^ab^	3.43 ± 0.32 ^a^
Non-pregnant	103	1.83 (1.59–2.12) ^a^	3.01 (2.69–3.37) ^a^	2.64 (2.34–2.98) ^a^	72.0 ± 3.07 ^b^	1453 ± 34.1 ^a^	4.77 ± 0.16 ^b^
*p*-value		<0.001	<0.001	<0.001	<0.001	0.004	<0.001

^a,b,c^ Means between rows with differing superscripts are different (*p* < 0.05); ^1^ Ewes were retrospectively allocated to the categories at the weaning of their first lambs (d465); ^2^ Was identified as pregnant at d301 but did not wean a lamb at d465.

## Data Availability

The data presented in this experiment are available within the article.

## Appendix A. Ewe Energy Requirement Equations

1. Ewe Maintenance [16]

$$ME_{b}=Species\times Sex\times 0.28\times EXP\;\left(-0.03\times Age\;in\;years\right)\times Predicted\;live\;weight^{0.75}\;/\;k_{m}$$

where:

*Species* = 1.0 for sheep,

*Sex* = 1.0 for females,

*k_m_* = M/D × 0.02 + 0.5,

where: M/D = energy concentration of the feed (MJME/kg DM) = 10.3 MJME/kg DM in this experiment (Litherland et al., 2002).

2. Ewe Liveweight Change [16]

$$ME_{g}=1.1\times LWC\times NE_{g}\;/\;k_{g}$$

where:

*LWC* = liveweight change (kg/d)

*NE_g_* = 0.92 × ((6.7 + (((920 × LWC)/(4 × SRW^0.75^)) − 1)) + (20.3 − (((920 × LWC)/(4 × SRW^0.75^)) − 1))/(1 + EXP (–6 × ((predicted live weight/SRW) − 0.4))))

SRW = mature body size = 65 kg for Romney,

*k_g_* = (M/D × 0.042) + 0.006,

*k_g_* (lactating) = 0.95 × ((M/D × 0.02) + 0.4).

3. Ewe Pregnancy Requirement [16]

$$\begin{array}{cc} ME\_p=(Total\; & birth\;weight/4)\times ((EXP(7.64\\ & -11.46\times \left(EXP\left(-0.00643\times day\;of\;pregnancy\right)\right))\times 0.0737\times EXP(-0.00643\\ & \times day\;of\;pregnancy))/kp \end{array}$$

where: *kp* = 0.133.

4. Lactation Requirements [16,21]

Ewe metabolizable energy (*ME*) required to produce milk (*Ewe lactation*)

$$Ewe\;lactation=\left(\left(\left(0.328\times 8.0\right)+\left(0.0025\times day\;of\;lactation\right)+2.203\right)\times daily\;milk\;yield\right)/\;k_{l}$$

where: daily milk yield was predicted from Peart, Edwards, and Donaldson [24] based on days of lactation and the number of lambs reared

$$k_{l}=0.95\;\times \;((M/D\;\times \;0.02)+0.4).$$

Lamb net energy (NE) requirement for maintenance of each lamb (*LmNEm*)

$$LmNEm=Species\times Sex\times 0.28\times EXP\;\left(-0.03\times age\;in\;years\right)\times predicted\;live\;weight^{0.75}$$

where:

*Species* = 1.0 for sheep,

*Sex* = 1.075 for lambs.

Lamb NE requirement for growth of each lamb (*LmNEg*)

$$\begin{array}{cc} LmNEg=0.92 & \times (\left(6.7+\left(\left(\left(920\times LWC\right)/\left(4\times {\left(SRW\times 1.2\right)}^{0.75}\;\right)\right)-1\right)\right)+(20.3\\ & -\left(\left(\left(920\times LWC\right)/\left(4\times {\left(SRW\times 1.2\right)}^{0.75}\right)\right)-1\right))\;/\;(1\\ & +EXP\;(-6\times \left(\left(predicted\;live\;weight/\left(SRW\times 1.2\right)\right)-0.4\right))))))\times 1.1\times LWC \end{array}$$

where:

*LWC* = liveweight change (kg/d)

*SRW* = 65 kg

The proportion of the lamb requirement met by DE in milk (*%MilkDE*)

%MilkDE=(0.26−(0.015×day of lactation / 7)) / 0.23

If day of lactation = 0, %*MilkDE* = 0.

If %*MilkDE* > 1, %*MilkDE* = 1.

Proportion of the lamb NE requirement for maintenance and growth met by milk (*%MilkNE*)

$$\begin{array}{cc} \%MilkNE= & \%MilkDE\times \left(\left(LmNEm/\left(0.9\times 0.82\right)+\left(LmNEg/\left(0.7\times 0.82\right)\right)\right)\right)\;/\;((\left(LmNEm/\left(0.9\times 0.9\right)\right)\\ & +\left(LmNEg/\left(0.7\times 0.9\right)\right))+(\%MilkDE\times ((\left(LmNEm/\left(0.9\times 0.82\right)\right)\\ & +\left(LmNEg/\left(0.7\times 0.82\right)\right))-\left(\left(LmNEm/\left(0.9\times 0.9\right)\right)+\left(LmNEg/\left(0.7\times 0.9\right)\right)\right))))) \end{array}$$

The proportion of the lamb ME requirement for maintenance and growth met by pasture (*LmMEp*)

$$LmMEp=\left(\left(LmNEm/k_{m}\right)+\left(LmNEg/k_{gain}\right)\right)\times \left(1-\%MilkNE\right)$$

where:

*k_m_* = (M/D × 0.02) + 0.5,

*k_gain_* = 0.7.

5. Total Ewe Daily Requirement

Total daily energy requirement = ewe maintenance + ewe liveweight change + ewe pregnancy + ewe lactation + LmMEp
